# The complete mitochondrial genome of a Dokdo shrimp, *Lebbeus groenlandicus*

**DOI:** 10.1080/23802359.2019.1693290

**Published:** 2019-11-21

**Authors:** Jungeun Kim, Jae-Pil Choi, Hui-Su Kim, Yejin Jo, Won Gi Min, Seungshic Yum, Jong Bhak

**Affiliations:** a Personal Genomics Institute, Genome Research Foundation, Cheongju, Republic of Korea;; b Korean Genomics Center (KOGIC), Ulsan National Institute of Science and Technology (UNIST), Ulsan, Republic of Korea;; c Ecological Risk Research Division, Korea Institute of Ocean Science and Technology (KIOST), Geoje, Republic of Korea;; d Ulleungdo-Dokdo Ocean Science Station, Korea Institute of Ocean Science and Technology (KIOST), Ulleung, Gyeongbuk, Republic of Korea;; e The Faculty of Applied Ocean Science, University of Science and Technology (UST), Geoje, Republic of Korea;; f Department of Biomedical Engineering, School of Life Sciences, Ulsan National Institute of Science and Technology (UNIST), Ulsan, Republic of Korea;; g Clinomics Inc., Ulsan, Republic of Korea

**Keywords:** *Lebbeus groenlandicus*, complete mitochondrial, Hippolytidae, Decapoda

## Abstract

*Lebbeus groenlandicus* is a shrimp species indigenous to the Dokdo islands in the East Sea of Korea. We report the 17,399 bp mitochondrial genome (mitogenome) of the species that consists of 13 protein-coding genes, 22 transfer RNAs (tRNAs), 2 ribosomal RNAs (rRNAs), and a control region (CR). A maximum-likelihood tree, constructed with 18 prawn and 45 shrimp mitogenomes, confirmed that *L. groenlandicus* occupies the most basal position within the Caridea infra-order and is closely related to Pandalidae shrimps.

Carideans, shrimp-like decapods, account for the majority of the marine species including more than 3100 species (Fransen and Grave 2009) found from the tropical to polar regions. *Lebbeus* is a hippolytid shrimp belonging to the infra order Caridea which consists of 68 different species. Three species of this genus, *L. grandimana* (Kim et al. 2010), *L. comanthi* (Lee et al. 2013), and *L. unalaskensis* (Kim et al. 2007), were previously identified in the South Sea of Korea. In this study, we analyze the mitogenome of *L. groenlandicus* and report another *Lebbeus* species, collected from the coast of the Dokdo islands in Korea (N37˚08′00.00″ E132˚02′00.00″).

The total DNA of *L. groenlandicus* was extracted and processed according to the previously described method (Kim et al. 2019). The voucher specimen is deposited at the Library of Marine Samples, KIOST, Geoje 53201, Republic of Korea (No. B-S-MA-00026777). We constructed whole-genome shotgun libraries using a TrueSeq library sample prep kit (Illumina, CA, USA) and sequenced them with the Illumina NovaSeq 6000 sequencer (Illumina, CA, USA). The mitochondrial genome of the *L. groenlandicus* was assembled with the NOVOPlasty (ver. 3.0), organelle genome assembler (Dierckxsens et al. 2017). We predicted the mitochondrial genes with the MITOS program (Bernt et al. 2013). A phylogeny analysis was conducted using the IQ-Tree webserver (Trifinopoulos et al. 2016) which uses a maximum-likelihood method.

The complete mitochondrial genome of *L. groenlandicus* is 17,399 bp in length (GenBank accession number: MN577077) and its GC ratio is 35.22%. It contains the typical gene set of 13 protein-coding, 22 tRNA and 2 rRNA genes, and a control region (CR). The gene order of *L. groenlandicus* was identical to the ones of Decapoda mitochondria.

To infer the phylogenetic relationships, we performed a maximum-likelihood analysis using the concatenated sequences of 13 protein-coding genes from 62 complete mitogenomes of various decapods, including 18 Dendrobranchiata and 45 shrimps in Pleocyemata sub-orders. The phylogenetic tree showed *L. groenlandicus* occupies the most basal position within the infra order Caridea and is closely related to the Pandalidae shrimp (Figure 1).

**Figure 1.**
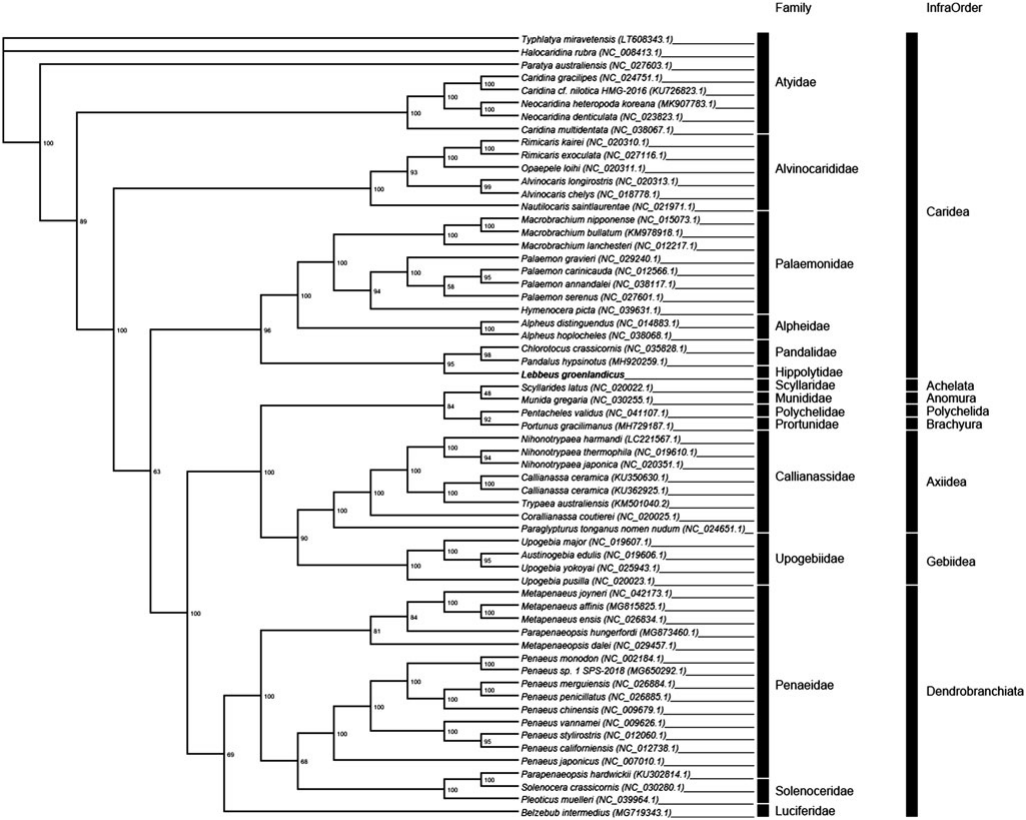
The phylogenetic tree of 62 species in Decapoda. The complete mitogenomes were downloaded from GenBank and the phylogenetic tree was constructed by a maximum-likelihood method with 1000 bootstrap replicates.
